# Collaboration between nurses and doctors in the decision‐making process when considering ending the life‐prolonging treatment of intensive care patients

**DOI:** 10.1002/nop2.1305

**Published:** 2022-08-20

**Authors:** Kristine Gjessing, Simen A. Steindal, Monica Evelyn Kvande

**Affiliations:** ^1^ Lovisenberg Diaconal University College Oslo Norway; ^2^ Division of Surgery Akershus University Hospital (Ahus) Lørenskog Norway; ^3^ Faculty of Health Studies VID Specialized University Oslo Norway; ^4^ Department of Anaesthesiology and Surgery University Hospital of North Norway Tromsø Norway

**Keywords:** ICU, interprofessional collaboration, nurse–physician relationship, withdrawing life‐prolonging treatment

## Abstract

**Aims:**

The aim of the study was to explore intensive care nurses’ collaboration with doctors’ when considering ending the life‐prolonging treatment of patients in the intensive care unit.

**Design:**

A qualitative method with an explorative descriptive design was employed.

**Methods:**

Data were collected through semi‐structured interviews with four intensive care nurses and four doctors working in three intensive care units at two university hospitals and one local hospital. The data were analysed using systematic text condensation. This study was reported according to the consolidated criteria for reporting qualitative research checklist.

**Results:**

Two categories were identified in the data analysis: listening to each other during the decision‐making process and continuity and having time to facilitate regular discussions of prognosis and treatment plans.

## 1 INTRODUCTION

The intensive care unit (ICU) patient’s condition is unpredictable and can shift rapidly from improvement to deterioration. Mortality rates in the ICU have been reported to be between 10% and 30% (Efstathiou et al., 2020). Although the severity of diseases in ICU patients is increasing, scientific and technological advances in intensive care medicine have improved survival rates (Vincent et al., 2017, 2018). A patient’s death can occur unexpectedly despite receiving a full life‐prolonging treatment or after a decision to withhold or withdraw treatment has been made, which studies showed to occur in about 42%–66% of the cases (Efstathiou et al., 2020).

Medical treatment decisions aim to return patients to a condition with an acceptable quality of life or to minimize suffering if survival is not possible. Healthcare professionals must aim to make a shared decision about treatment, and the patient’s opinion must be included, if possible (Michalsen et al., 2019; Myburgh et al., 2016). Studies have shown that good communication and collaboration are required to achieve consensus among the interdisciplinary treatment team and to ensure the best possible transition from curative to palliative care in the ICU (Brooks et al., 2017; Metaxa et al., 2021). The quality of interprofessional collaboration is influenced by organizational, relational, processual and contextual factors, such as culture, professional power, rituals and routines (Kendall‐Gallagher et al., 2017; Reeves et al., 2018).

## 2 BACKGROUND

Interprofessional care in the ICU is described as “care provided by a team of healthcare professionals with overlapping expertise and an appreciation for the unique contribution of other team members as partners in achieving a common goal” (e.g. nurses, doctors and other healthcare professionals) (Donovan et al., 2018; Michalsen et al., 2019). Doctors and nurses constitute the ICU team, supported by doctors and professionals from other specialties (Donovan et al., 2018; Ervin et al., 2018).

Interprofessional shared decision‐making is a collaborative process among clinicians that allows for shared decisions about important questions (Michalsen et al., 2019). A lack of understanding or disagreements in the treatment team during decision‐making about a life‐prolonging treatment can induce moral stress, which may negatively impact the emotions of the patients and the individuals on the team (Henrich et al., 2017; Paddley et al., 2022). Dodek et al. (2016) found that moral stress was more prominent in nurses and other health professionals than in doctors in the ICU. These differences may be due to the hierarchy in the organization, lack of opportunity for collaboration, different understandings of each other’s roles or individual differences and cultures.

Previous studies have suggested that ICU nurses experience little involvement, that doctors do not always acknowledge their views or assessment of patient cases and that ICU nurses could feel ignored in decision‐making discussions (Flannery et al., 2020; Halvorsen, 2017; Taylor et al., 2020). A study showed that even though doctors said that they also include input from nurses in their decisions about end‐of‐life care, nurses did not feel included (Flannery et al., 2020). A literature review found that experienced physicians preferred the inclusion of nurses in the end‐of‐life decision‐making process, which facilitated a cohesive approach to decision‐making and enhanced satisfaction among doctors and nurses (Flannery et al., 2016). Kvande et al. (2017) found that doctors should increase their willingness to listen to nurses’ observations and opinions. Although doctors are responsible for the final decision‐making, ICU nurses should be informed of their right to partake in the decision‐making process about patient treatment.

It is well established in the literature that good communication is a prerequisite to ensure that the transition to final treatment is performed in the best possible way in an ICU and that this is often perceived as problematic (Brooks et al., 2017; Kendall‐Gallagher et al., 2017; Myburgh et al., 2016). Additionally, the process of improving routines for interdisciplinary collaboration in an organization is complex but important (Kendall‐Gallagher et al., 2017). Previous research has focused mostly on communication among the treatment team and relatives (Chen et al., 2018; Curtis et al., 2016; Jensen et al., 2017; Nygaard et al., 2022) or only on nurses’ experiences (Kendall‐Gallagher et al., 2017; Taylor et al., 2020). However, few previous studies have explored ICU nurses’ and doctors’ experiences with communication and collaboration with one another in the decision‐making process when considering ending the life‐prolonging treatment of patients in the ICU. Therefore, the aim of the present study was to explore ICU nurses’ and doctors’ experiences about collaboration with one another when considering ending the life‐prolonging treatment of patients in the ICU.

## 3 METHODS

### 3.1 Design

This study used a qualitative method with an exploratory descriptive design to collect data from semi‐structured interviews with ICU nurses and doctors. This design is suitable for exploring the participants’ experiences when considering ending the life‐prolonging treatment of patients in the ICU (Hunter et al., 2019). In qualitative description, the researchers aim to achieve a low level of interpretation and stay close to the data and to the surface of words and events (Sandelowski, 2000). The study was reported according to the consolidated criteria for reporting qualitative research checklist.

### 3.2 Participants and recruitment

The informants were recruited from three ICUs at two university hospitals and one local hospital in Norway. The ward managers recruited informants by sending out emails containing information about the study and using purposeful sampling to select rich and varied participants. The following inclusion criteria were applied: being either an ICU doctor or an ICU nurse, having worked in the ICU ward for at least 2 years and having experience in participating in a treatment team during the decision‐making process of terminating the life‐prolonging treatment of ICU patients over 18 years. Those who agreed to participate contacted the first author directly. The study sample comprised four ICU nurses and four doctors. There were three female and one male ICU nurses and two female and two male doctors. The doctors had a mean age of 54 years and 21 years of ICU experience, while the nurses had a mean age of 45 years and 17 years of ICU experience.

### 3.3 Data collection

Semi‐structured interviews were conducted with all participants between January and March 2021 and lasted 40–70 min. The interviews were conducted digitally via video calls and audio‐recorded, and the participants chose a convenient time and location for the interviews. An interview guide with open‐ended questions was used to facilitate reflection and dialogue with the participants. This covered topics such as participants’ thoughts on their roles in the teams, requests to describe specific situations with good and bad collaboration and communication, routines for collaboration in the ward, what the participants deemed important in the decision‐making process and possible follow‐up questions on each topic (Appendix).

### 3.4 Analysis

The first author (KG) transcribed the interviews verbatim shortly after each interview to become well acquainted with the data material. The data material was analysed using systematic text condensation (Malterud, 2012). This involved conducting an inductive and thematic cross‐case analysis of the data material. In the first step, all transcripts were read multiple times to get an overview, and eight preliminary themes were identified. In the second step, meaning units were found, coded and organized into code groups, and the eight preliminary themes were eventually transformed into three code groups. In the third step, the meaning units in each of the three code groups were organized into subgroups. Furthermore, the meaning units in each subgroup were condensed and abstracted into condensates. In the fourth step, each condensate was developed into an analytic text. An example of the analysis process is shown in Table 1.

**TABLE 1:** Example of stepwise analysis from meaning units of meaning to categories using systematic text condensation (STC)

Unit of meaning	Subgroup	Category
It works so well when the doctor is open and responsive to our opinions, and I feel many doctors are, but some are not. I feel it is because we are different as individuals. But I feel like they also think it feels good and safe when we support each other. (Nurse 3) I think it is written in the law that the chief physician is responsible to make the decision and stand by it, but he or she also have a duty to gather and use the information available from the nurses in the team. (Doctor 1) It is important that we support each other. In the decision‐making process, it is important to involve as many voices as possible, as the medical group must support each other. During difficult treatment courses, I have found that collaboration between nurses and doctors may become even better because we form a united front together. (Doctor 4) The doctors can include us, and listen to what we say, but then they don’t take any further note of what we say. If only they would not just say “Yeah, I heard what you said, now you can return to the patient,” but that our opinions and thoughts would actually be taken into account when making a decision. (Nurse 2) I’m very happy to get help from the nurses, and listen to their observations and thoughts, because I see that it helps me. But I know that not everyone understands that benefit. It might be because I consider myself to be confident in my position, and don’t need to hide behind a mask. I mean, I’m not afraid that the nurses won’t respect me as a professional because I ask them for help. (Doctor 3) The nurse is also in the team to convey her thoughts on the medical issues as far as she is competent to do. And I feel like the issues regarding medical ethics, or ethics in general, is something many doctors should be told they don’t have monopoly on knowing anything about. (Doctor 4)	Stand together as a united team Including the nurses’ opinions and observations in the decision‐making process	Listening to each other during the decision‐making process

### 3.5 Trustworthiness

KG works as an ICU nurse and has previous experience with end‐of‐life treatment and the decision‐making process in other areas of nursing. KG does not work at either of the wards where participants were recruited, which ensured that what the participants said in the interviews was not affected by personal relations with the first author.

KG strengthened reflexivity by making notes about preconceptions related to the study aim. This created awareness of the potential prejudices in the theme and enhanced the ability to be open and receptive to new perspectives and experiences, as told by the participants. To ensure the credibility of the results, preconceptions were also challenged through discussions with the second author (SAS) and the last author (MEK). Furthermore, the data were analysed by KG, while SAS and MEK asked critical questions throughout the analysis process to help uncover alternate interpretations. All authors agreed on the final categories.

The interview guide was discussed with SAS and MEK to ensure its relevance to the study aim. During the interviews, KG’s interpretations of what the participants shared were validated by summarizing and asking questions about what they said to ensure that there were no misunderstandings.

Credibility was enhanced by purposeful sampling from ICUs in three different hospitals, including participants from both university hospitals and a local hospital. This ensured that the participants with varied experiences clarified different aspects related to the aim of the study.

Transferability was facilitated through a description of the context, participants, data collection, analysis process and rich descriptions of the results with relevant quotes. Such descriptions are important to enable the reader to consider whether the findings can be transferable to their context (Graneheim & Lundman, 2004).

### 3.6 Ethical considerations

The Norwegian Center for Research Data (reference number 194187) and the research ethics committees at each hospital approved the study. All participants received written information about the study in advance, along with the opportunity to withdraw and safeguard their privacy and anonymity. Written informed consent was collected from all participants before the interviews.

## 4 RESULTS

Two categories were identified from the data analysis: listening to each other during the decision‐making process and continuity and having time to facilitate regular discussions of prognosis and treatment plans. Table 2 provides an overview of the identified categories and their subgroups.

**TABLE 2:** Overview of categories and subgroups

Categories	Subgroups
Listening to each other during the decision‐making process	Stand together as a united team
	Including the nurses’ opinions and observations in the decision‐making process
	Doctors and nurses have different views of the patients
Continuity and having time to facilitate regular discussions of prognosis and treatment plans	Continuity as a prerequisite for good collaboration around the case
	Regular meeting points and time for team discussions
	Making plans and having prognosis discussions early in the patient’s course of illness

### 4.1 Listening to each other during the decision‐making process

Doctors stated that good collaboration required that both parties were prepared to listen and that arguments were well founded. However, doctors tended to control the decision‐making processes and highlighted the need to involve nurses earlier. Nurses stated that the team could have had more constructive treatment discussions if nurses had been involved earlier. Doctors said the importance of having support in the team and reported that, in challenging situations, togetherness could be improved by forming a “united front” to solve the issue. Nurses stated that they had good cooperation routines when a patient was admitted to the ICU but that these disappeared when the treatment was headed towards termination.

We work so well together as a team in the reception of a patient when the focus is to save lives. However, to maintain this when you choose to change focus to end treatment … It doesn’t mean that communication and cooperation should stop. (Nurse 3)

Nurses said the need to be included and listened to by doctors in the discussions about the ending life‐prolonging treatment of the patients. However, since nurses could not leave the patient rooms and sometimes said unwanted in morning meetings with the doctors, nurses seldom participated in such discussions. Consequently, the doctor’s decisions often came as a surprise. Nurses acknowledged that the doctor has the legal responsibility but considered their bedside perspective to be essential in the decision‐making process. Furthermore, nurses said rejected by the doctor when they spoke their opinion, which made them question their own opinions, judgements and experiences.

I regularly asked these questions if we should continue treatment, but no one reflected on what I asked, just quickly replied, ‘No, of course we will continue’. (Nurse 3)

Nurses reported challenges in communicating clinical head‐to‐toe assessments made about small changes in the patient’s condition and using professional language that helped to present their observations and thoughts on patient prognosis to the attending doctor.

We nurses should be tougher, trust our knowledge, and be proud in our profession and choices. We do not need to give up but communicate our observations clearly to doctors. […] (Nurse 2)

Doctors stated that nurses do not hesitate to make contact in general but that they were quite cautious and unclear about their intentions with the conversations they initiated.

In my experience, the nurses initiate the discussion quite carefully by asking questions like ‘What do you think? How do you feel about this?’. They do not state directly that their opinion is that the treatment should be ended. (Doctor 1)

Nurses reported that many doctors hesitated to initiate ethical discussions about ending the life‐prolonging treatment of ICU patients. Doctors stated that ethics is an important area, but they experienced that nurses were better at focusing on ethical issues and more realistic about prognosis than they were. Nevertheless, some doctors described that nurses’ closeness to the patient over time made them physically and mentally exhausted, complicating their ability to make objective assessments when considering treatment termination.

The nurses work closely with the patients over a long time, which can be physically and emotionally tiring […] They can get too close to the patient, and it can therefore be easier for us doctors to be objective. (Doctor 4)

Doctors said that they often discussed patient cases with each other long before consulting nurses; thus, nurses’ opinions did not have real significance. Although doctors admitted to knowing they had a legal obligation to use all available information sources, including nurses, to gather information for a comprehensive assessment basis, this was not done adequately. Lack of time was mentioned as an important barrier.

Nurses reported different personalities and relationships as influencing factors to whether they found it easy to raise difficult issues with the doctors. Nurses’ good experiences with discussions about ending life‐prolonging treatment were when the doctor was responsive, open and appeared experienced and confident. Nurses explained that when doctors seemed inexperienced and insecure, they did not take the time to listen to them.

We tried different things, such as checklists with topics to go through with the doctor, but it doesn’t matter how many checklists you have if they aren’t interested in a dialogue. (Nurse 2)

One doctor talked about a situation in which the nurse was reluctant with her suggestions, based on her previous experience that such suggestions were not welcome:

It would have helped both me, to lead the situation, and the patient! We must appreciate it when nurses present a suggestion, but that’s not always the case. (Doctor 3)

### 4.2 Continuity and having time to facilitate regular discussions of prognosis and treatment plans

Both nurses and doctors identified continuity as a prerequisite for collaboration in the decision‐making process about treatment. Nurses highlighted that doctors who were familiar with the ward were better at facilitating team meetings to discuss prognosis and listened to other team members’ thoughts and suggestions about treatment. However, nurses stated that there were often new doctors in charge of the patients. Doctors, on the contrary, said that a lack of nurse continuity hindered involving nurses in decisions and highlighted the value of working with well‐known nurses who could provide useful information about patients.

I’m very happy to get help from the well‐known nurses and listen to their observations and thoughts because I see that it helps me. But I know that not all of my colleagues understand that benefit. It might be because I consider myself to be confident in my position. (Doctor 3)

Early discussions when considering ending life‐prolonging treatment of ICU patients were valued by both nurses and doctors. Improving early documentation of treatment escalation plans could create security and make it easier for new colleagues to familiarize themselves with the situation and make assessments of the patient.

It’s a security for both nurse, doctor, and patient that a treatment plan has been discussed in structured and orderly forms. The situation can change, of course, but then a basic idea has been documented and clarified, and that’s a security for everyone. (Nurse 4)

One thing we want to achieve, but is difficult to implement, is to assess all patients for the life‐prolonging treatment within 72 hours of admission. Currently, I can’t say we’re able to do that, but it would be ideal. (Doctor 4)

Assessments about life‐prolonging treatment should be followed up with interdisciplinary meetings to provide a timed perspective for new assessments of prognoses. Doctors pinpointed that an early assessment of resuscitation status should be established as a routine. The most unpleasant situation for both nurses and doctors was when the interdisciplinary team disagreed about prognosis and failed to create a treatment plan in time.

Participants agreed that regular team meetings usually occurred only 2 weeks after the patient’s admission and that this was not well established as a routine. Nurses and doctors said difficulties in finding time for these meetings, and it was mostly up to the individual nurse to organize. However, routines and interdisciplinary meetings around patients who received extracorporeal membrane oxygenation (ECMO) treatment are well established.

Everyone is present in the daily ECMO meetings, and there is a good dialogue and common understanding in the team throughout the treatment course. With other patients, this routine has somehow been reorganized outside the patient’s room. (Nurse 2)

Nurses and doctors highlighted the need for a daily meeting together outside the patient’s room, where they would not be interrupted to discuss and plan treatment and prognosis and explain their viewpoints to one another. Doctors said that this could improve their ability to see the patient from nurses’ perspective. Both nurses and doctors reported that although the ward routines were good on paper, there was insufficient time to organize meetings. Nurses reported inexperienced and stressed doctors as factors that made meetings difficult to implement.

The doctors don’t have enough time; they’re always in a hurry, and they’re too few for the number of patients we have. […] Some are not very experienced, which makes them insecure and impatient to listen to nurses. (Nurse 2)

## 5 DISCUSSION

This study aimed to explore ICU nurses’ and doctors’ experiences about collaboration with each other when considering ending the life‐prolonging treatment of patients in the ICU. Two categories were identified: listening to each other during the decision‐making process and continuity and having time to facilitate regular discussions of prognosis and treatment plans.

Nurses experienced challenges in communicating their observations and thoughts about the patient’s condition in a professional language that made doctors understand and consider their inputs. Doctors experienced nurses as being unclear about their opinions when discussing the patients. Kvande et al. (2017) underscore the need to strengthen nurses’ ability to report their clinical observations and interpretations to doctors on shift. According to Benner et al. (2011), learning how to effectively communicate clinical interpretations requires the ability to think clearly while in an ongoing situation and name small changes that can indicate a transition in the patient’s condition. Previous studies showed that communication tools/checklists, team training about team communication and multidisciplinary structured work shift evaluation are effective in improving nurse–doctor relationships and communication skills. This could positively impact patients’ treatment, outcomes and experiences in the ICU (Wang et al., 2018; Zamanzadeh et al., 2020).

In this study, doctors said that they tended to discuss patient cases among themselves without involving nurses in discussions about ending life‐prolonging treatment. This paralleled nurses’ experiences, who said excluded or not listened to by the doctors. When nurses are excluded from participating in discussions, they may not know and understand the basis for the decisions made and have little opportunity to promote their viewpoints, which could contribute to a more holistic approach to treatment (Halvorsen, 2017). Following Benner et al. (2011), the interpretation of a situation is social, and clinical reasoning requires all clinicians to clearly and openly reason about the changes in that particular situation. Similar with our study, Zamanzadeh et al. (2020) found that barriers for nurse participation in multidisciplinary ward rounds were lack of time and inconvenient or lack of non‐existent physical space to conduct the meetings with the doctors. Furthermore, for nurses to tell doctors about their observations about subtle changes in the patient’s condition, they need to experience respect and trust from doctors and that they are willing and interested in listening to them (Benner et al., 2011). In line with the study of Kvande et al. (2017), our participants also promoted a need for better knowledge and awareness among nurses about their rights and roles in the decision‐making process. Together, these findings reveal a need to identify barriers to nurse participation in the organizations and to investigate nurses’ skills and knowledge about their legal rights to participate in the decision‐making process.

Interestingly, both doctors and nurses in this study stated that less experienced doctors appeared less open and confident in the context of listening to nurses’ opinions and that seasoned doctors appeared calmer and more confident in the collaboration with nurses. This raises concerns about whether those with less experience collect knowledge from fewer team members and professions and make decisions on a narrower knowledge basis than those with more experience. Benner et al. (2011) state that getting to know team members’ talents and skills is knowledge that develops with experience over time, and that using this knowledge makes a statistically significant difference in boosting the team’s function. To improve interprofessional collaboration, it is necessary to understand how relational, organizational, procedural and contextual factors impact such collaboration (Kendall‐Gallagher et al., 2017; Wang et al., 2018). This suggests that ward managers should consider these factors and investigate how routines for collaboration and team building can be improved to use all available competences in the treatment team.

In this study, both doctors and nurses perceived continuity as a prerequisite for collaboration in the decision‐making process when considering ending life‐prolonging ICU treatment. However, both professions often experienced collaboration challenges due to a lack of continuity. Benner et al. (2011) stated that for team members to present a clinical interpretation of each other, they need to achieve a good overview of the patient’s clinical history, trajectory and previous responses to treatment. The ability to gain this overview was enhanced by continuity among the staff, and a lack of continuity was explained to prompt fragmented care, such as the interruption that moving a patient to a different ward or level of care can cause (Benner et al., 2011).

Our participants valued early planning discussions and highlighted that a documented treatment escalation plan created security for the entire treatment team. Such plans could form an explained and warrantable information basis about the patient for future decision‐making if the patient’s condition deteriorated, where the patient also has a chance to document their preferences, thus improving patient care and outcomes (Lightbody et al., 2018; May et al., 2020). Still, the process of identifying the illness trajectory and setting agreed‐upon goals for treatment often happens too late in a patient’s treatment course (Lightbody et al., 2018). This was mentioned as an issue by both professions in this study, and they revealed that these situations are the most unpleasant for the treatment team. Ma et al. (2019) found that early palliative care consultation (in 48 h of ICU admission) induced more effective treatment discussions and consequently increased the transition to Do‐Not‐Resuscitate code status. This made for faster implementation of palliative care or discharge to palliative care units, which in turn decreased ICU and post‐ICU resource utilization and costs. Combined, these findings indicate that improving routines for early assessments and screening of the patients’ conditions and mortality/morbidity risk when admitted to the ICU could improve the effectiveness and quality of interdisciplinary collaboration and benefit the whole organization on an administrative level.

### 5.1 Study limitations

The small sample size of four ICU nurses and four doctors could limit our study since there could be experiences that we have not been able to identify. However, our participants spoke openly and shared their experiences during the interviews, and the study’s aim was considered narrow. Consequently, the sample size was deemed to have provided sufficient information power (Malterud et al., 2016). Additionally, most of the participants had extensive experience in the field and at their workplace and less experienced participants may have provided different knowledge.

International perspectives on ICU settings, work routines and the decision‐making hierarchy can vary, which could limit transferability. However, this study provides an example of experiences in the subject. Another limitation may be that most of the doctors were from the same hospital. However, the doctors noted that they had experience in several hospitals in different parts of the country and even abroad. This gave them extensive and varied experience in the field.

## 6 CONCLUSION

The study emphasizes the need to identify and remove possible barriers to ICU nurses’ involvement in treatment discussions and to improve nurses’ professional language to make them more confident in their role and better at explaining their opinions to the doctors. Both doctors and nurses should be more aware of ICU nurses’ rights to be included in the decision‐ making processes about life‐prolonging treatment. There is a need for ward managers to consider organizational factors and routines to improve team building to lower the threshold for using all competences in the team. In addition, both nurses and doctors experienced that continuity among both professions improved the quality of the treatment and created security for patients, family and treatment teams throughout the patient’s illness trajectory. This enabled the team to achieve a more detailed overview of the patient’s clinical history, which, in turn, could improve team collaboration and make for a more holistic treatment.

### 6.1 Relevance to clinical practice

The study findings reveal a need to improve ICU nurses’ professional language when reporting to doctors and knowledge about their rights to be involved in treatment discussions. Additionally, possible barriers to nurses’ involvement in wards should be identified and removed. Ward managers should consider organizational factors and develop good routines to improve team building and lower the threshold for using all available competences on the team.

## AUTHORS’ CONTRIBUTIONS

First author: Kristine GJESSING (KG), RN, Intensive Care Nurse Specialist, MNSc. Second author: Simen A. STEINDAL (SAS), RN, PhD, Professor. Third author: Monica Evelyn KVANDE (MEK), RN, Intensive Care Nurse Specialist, MNSc, PhD. KG and MEK: Study design and interview planning. KG: Interview performance. All authors (KG, SAS and MEK) participated in the analyses of the collected and transcribed data, discussed the relevance of the results, drafted the manuscript together, and have read and approved the final manuscript.

## CONFLICT OF INTEREST

The authors declare no conflict of interest.

## FUNDING INFORMATION

None.

## Data Availability

Research data are not shared.

## Interview guide

Please note that this guide represents only the main themes to be discussed with the participants and does not include the various prompts that may also be used for each question. Follow‐up questions will also be used for each topic, such as “Can you please tell me a little bit more about that?” and “What does that look like for you?”

### Introduction

Thank you for agreeing to participate in this interview. I am interviewing you to better understand the experiences of intensive care unit (ICU) nurses and doctors about communication and collaboration with one another when considering ending the life‐prolonging treatment of patients in the ICU. Hence, there are no right or wrong answers to any of my questions. I am interested in your own experiences.

Depending on how much information you would like to share, the interview should take approximately 1 h. With your permission, I would like to audio record the interview so that I do not miss any of your comments. All responses will be kept confidential. This means that your interview responses will only be shared with the research team members, and we will ensure that any information we include in our report does not identify you as the respondent. You may decline to answer any question or stop the interview at any time and for any reason.

Are there any questions about what I have just explained?

May I turn on the digital recorder?

### Establishing rapport

Before we begin, it would be nice if you could tell me a little bit about your background as an ICU nurse/doctor.

Can you tell me what experiences you have had with participating in the decision‐making process related to the withdrawal of life‐prolonging treatment in ICU patients?

What do you consider important in the decision‐making process for the withdrawal of life‐prolonging treatment in ICU patients?

What are your thoughts regarding nurses’ and doctors’ roles in the decision‐making process for the withdrawal of life‐prolonging treatment in ICU patients?

Could you describe a situation in which the collaboration between ICU doctors and ICU nurses worked well?

What worked well?

Could you describe a situation in which the collaboration between ICU doctors and ICU nurses did not work well?

What did not work well?

Who takes the initiative to discuss the withdrawal of life‐prolonging ICU treatments?

Do you have any routines, collaboration meetings, or common meetings in which you discuss whether treatments should be continued?

If yes, who is included in the team?

How do you think the team is functioning?

Is there something you believe would enhance collaboration between doctors and nurses in the team?

How do you perceive communication in the team regarding the process of withdrawing life‐prolonging ICU treatment?

Could you describe a situation in which communication in the team was good?

Could you describe a situation in which communication in the team was poor?

Could you describe a situation in which communication in the team was challenging?

What do you think is the biggest cause of poor communication and collaboration and/or challenges that arise?

Is there something you have experienced that has particularly enhanced or created opportunities for good communication in the team?

Have you ever experienced that patient characteristics, circumstances, and/or backgrounds (age, diagnosis, culture, etc.) have influenced the team’s communication and collaboration?

### Conclusion

Is there anything else that you would like to comment on that I have not already asked you about?

Thank you very much for your time and for the information you shared today.
